# A genotype-directed phase I–IV dose-finding study of irinotecan in combination with fluorouracil/leucovorin as first-line treatment in advanced colorectal cancer

**DOI:** 10.1038/bjc.2011.206

**Published:** 2011-06-07

**Authors:** E Marcuello, D Páez, L Paré, J Salazar, A Sebio, E del Rio, M Baiget

**Affiliations:** 1 Medical Oncology, Universitat Autònoma de Barcelona, Pare Claret 167, Barcelona 08025, Spain; 2 Genetics Departments, Hospital de la Santa Creu i Sant Pau, Universitat Autònoma de Barcelona, Pare Claret 167, Barcelona 08025, Spain; 3 U-705 CIBERER, Barcelona, Spain

**Keywords:** irinotecan, colorectal cancer, UGT1A1, pharmacogenetics

## Abstract

**Background:**

Infusional fluorouracil/leucovorin (FU/LV) plus irinotecan (FOLFIRI) is one of the standard first-line options for patients with metastatic colorectal cancer (mCRC). Irinotecan is converted into 7-ethyl-10-hydroxycamptothecin (SN-38) by a carboxylsterase and metabolised through uridine diphosphate glucuronosyl transferase (UGT1A1). The UGT1A1^*^28 allele has been associated with the risk of developing severe toxicities. The present trial was designed to define the maximum tolerated dose according to UGT1A1 genotype. This report focuses on the results of tolerance to different escalated doses of FOLFIRI first-line of chemotherapy.

**Patients and methods:**

Patients undergoing first-line treatment for mCRC and eligible for treatment with FOLFIRI were classified according to UGT1A1 genotype. A total of 94 patients were eligible for dose escalation of irinotecan. The starting dose of biweekly irinotecan was 180 mg m^−2^ for the ^*^1/^*^1, 110 mg m^−2^ for the ^*^1/^*^28 and 90 mg m^−2^ for the ^*^28/^*^28 genotypes.

**Results:**

The dose of irinotecan was escalated to 450 mg m^−2^ in patients with the ^*^1/^*^1 genotype, to 390 mg m^−2^ in those with the ^*^1/^*^28 genotype and to 150 mg m^−2^ in those with the ^*^28/^*^28 genotype. Neutropenia and diarrhoea were the most common grade 3 or 4 toxicities.

**Conclusions:**

Our results demonstrated that the recommended dose of 180 mg m^−2^ for irinotecan in FOLFIRI is considerably lower than the dose that can be tolerated for patients with the UGT1A1 ^*^1/^*^1 and ^*^1/^*^28 genotypes. The maximum tolerable dose (MTD) in patients with a high-risk UGT1A1 ^*^28/^*^28 genotype is 30% lower than the standard dose of 180 mg m^−2^.

Colorectal carcinoma is the third cause of cancer-related mortality in industrialised countries. Although some metastatic disease may benefit from surgical resection, chemotherapy is the prime therapeutic tool in advanced disease, as it aims to palliate symptoms and increase time to disease progression (TTP) as well as overall survival.

In the last 40 years, fluorouracil (FU) has been the most widely used chemotherapeutic agent in the treatment of advanced disease. In the early 1980s, a modulation of FU with folinic acid helped to increase the response rate and lengthen the TTP. Furthermore, the advent of new drugs such as irinotecan and oxaliplatin in different combinations with fluoropyrimidines has improved outcome in many patients (Douillard *et al*, 2000; Saltz *et al*, 2000; Köhne *et al*, 2005; Saunders and Iveson, 2006). This scenario has improved even more with the recent introduction of new biological drugs such as bevacizumab or cetuximab in the treatment schemes (Hurwitz *et al*, 2004; Van Cutsem *et al*, 2009).

Infusional FU/leucovorin (LV) plus irinotecan (FOLFIRI) is one of the standard first-line options for patients with metastatic colorectal cancer (mCRC). The recommended dose for irinotecan in combination with FU in first-line chemotherapy is 180 mg m^−2^ every 2 weeks (Ducreux *et al*, 1999; Tournigand *et al*, 2004), and a phase-II trial reported a high response rate using 260 mg m^−2^ every 2 weeks with a median TTP of 8 to 10 months and median survival of 22 months (Duffour *et al*, 2002). When doses of irinotecan were increased, the most frequent side effects were myelotoxicity and diarrhoea.

Irinotecan is converted into 7-ethyl-10-hydroxycamptothecin (SN-38) by a carboxylsterase, and finally metabolised through uridine diphosphate glucuronosyl transferase enzyme (UGT), predominantly by UGT1A1 isoenzyme. UGT1A1 is a polymorphic enzyme. The number of TA repeats (5, 6, 7 or 8) in the TATA box of the UGT1A1 promoter region is inversely correlated with the gene transcription efficiency and overall enzyme activity. The presence of seven repeats (TA_7_) results in the variant allele UGT1A1^*^28 compared with the normal genotype of six TA repeats (UGT1A1^*^1). This polymorphism has been related to myelosuppression and severe diarrhoeic toxicity in UGT1A1^*^28/^*^28 and UGT1A1^*^1/^*^28 genotypes (Ando *et al*, 2000; Innocenti *et al*, 2004; Marcuello *et al*, 2004; Rouits *et al*, 2004; Toffoli *et al*, 2006; Hoskins *et al*, 2007; Kim and Innocenti, 2007). Individualising doses according to UGT1A1 genotype has been proposed to optimise treatment efficacy and tolerance. The present trial was designed to define the maximum tolerated dose (MTD) according to UGT1A1 genotype. We report the results of tolerance to different escalated doses of irinotecan plus infusional 5-fluorouracil/leucovorin (FOLFIRI) in first-line chemotherapy.

## Patients and methods

### Patients

A total of 94 patients receiving the FOLFIRI regimen were included in the study. Eligibility criteria included histologically proven mCRC or a locally advanced recurrence after surgery, age ⩾18 years, an Eastern cooperative oncology group (ECOG) performance status of 0 to 2, life expectancy of more than 3 months and adequate bone marrow function (haemoglobin ⩾10 g dl^−1^, neutrophil count ⩾1500 *μ*l^−1^ and platelet count ⩾100 000 *μ*l^−1^), renal function (creatinine clearance more than 60 ml min^−1^) and liver function (serum creatinine and bilirubin <1.5 × the upper limit of normal and AST and ALT ⩽2.5 × the upper limit of normal). The exclusion criteria were ECOG>2, apparent jaundice or severe comorbidities. Analysis of the UGT1A1 genotype was mandatory for inclusion in this trial.

Before starting the treatment, complete medical history, physical examination, complete analytical studies including blood counts, liver and renal function, ionogram, lactate dehydrogenase, alkaline phosphatise, CEA determination and chest, abdomen and pelvic computed tomography scan were performed in all patients. Patients were classified according to the European Organisation for Research and Treatment of Cancer (EORTC) clinical model validated by Köhne *et al* (2002). Follow-up was conducted throughout the treatment period until disease progression or death. Responses and progression were evaluated every 12 weeks using Response Evaluation Criteria in Solid Tumours (RECIST). Time to disease progression was assessed from inclusion until disease progression was evident. Toxicity was assessed at every visit using the National Cancer Institute Common Toxicity Criteria version 3.0. Written informed consent was obtained and the study was approved by the Institutional Ethics Committee and by the Spanish Agency for Medicines and Medical Devices (N° EudraCT ‘2007-006788-65’).

### Study design and dose escalation

The primary end-point was assessment of the MTD of irinotecan and dose-limiting toxicity (DLT) at cycle 1. Secondary end-points included the objective response rate and TTP.

Patients were treated with the FOLFIRI scheme. The dose of irinotecan, administered as a 2-h intravenous (i.v.) infusion on day 1, was escalated at different levels according to UGT1A1 genotype. The starting dose of irinotecan was 180 in ^*^1/^*^1 patients; 110 in *1/^*^28 and 90 in ^*^28/^*^28.

Leucovorin was administered as a 200 mg m^−2^ in 2 h of i.v. infusion during irinotecan infusion followed by 5-FU at 400 mg m^−2^ bolus injection, and 5-FU 600 mg m^−2^ in 22 h infusion on days 1 and 2 (Douillard *et al*, 2000). Treatment was repeated every 14 days, if blood counts were adequate. One cycle of treatment consisted of two courses of chemotherapy in 28 days. A complete analytical study was carried out every 7 days during the first cycle.

Dose-limiting toxicity was defined when haematologic grade 4 toxicity, or non-haematologic grade 3–4 toxicities appeared during cycle 1 and persisted despite supportive measures (including antiemetics or antidiarrhoeal agents). Any kind of toxicity that caused hospitalisation or a delay of more than 2 weeks in the treatment administration was considered a DLT. Three patients were scheduled at each dose level. If no toxicity was detected, three new patients were treated with the following dose level. When one patient presented DLT, three new patients received that dose level, and if only one patient had DLT, the following patients were treated with the higher dose level. If two out of three or two out of six patients experienced DLT, the level below was considered MTD. Patients with DLT continued with a 20% dose reduction in the following cycles. Colony-stimulating granulocyte factor (FEC-G) was allowed in patients with grade 3–4 neutropenia.

### UGT1A1 genotyping assay

Genomic DNA was extracted from peripheral leukocytes by the salting-out procedure (Miller *et al*, 1989). The TA index of the UGT1A1 promoter was genotyped by fragment sizing. Polymerase chain reaction was performed in a total volume of 25 *μ*l containing template DNA (80 ng *μ*l^−1^), according to Monaghan *et al* (1996). The primers used were a forward primer that was modified by the addition of a 5′ fluorescent-labelled FAM and an unlabelled reverse primer (UGT-FAM_F; 5′-GTCACGTGACACAGTCAAAC-3′, UGT_R 5′-TTTGCTCCTGCCAGAGGTT-3′). The PCR product (TA^*^1, 98 bp; TA^*^28, 100 bp), the internal size standard and Hi-Di formamide (GeneScan 500, Applied Biosystems, Foster City, CA, USA) were mixed. The samples were then run in the ABI Prism 3100 Genetic Analyzer (Applied Biosystems). Fragment sizes were determined by comparison with the internal standard GeneScan 500 using the local Southern algorithm and analysed by GeneMapper software version 3.5 (Applied Biosystems).

Normal, heterozygous and homozygous sequenced samples were included on every run as a quality control. Genotypes were assigned based on the number of TA repeats in each allele (i.e., TA^*^1/^*^1, TA*1/TA^*^28 and TA^*^28/TA^*^28).

### Statistics

Differences between categorical variables were measured by the *χ*^2^-test. The Mann–Whitney test was used for comparisons between responses in UGT1A1 genotype and dose. To ascertain the effect of irinotecan dose and the UGT1A1 genotype on response rate, a logistic regression was used as a multivariate method after adjustment for other relevant clinical variables. Time to disease progression was assessed by the Kaplan–Meier method and log-rank test for comparisons. Results were considered statistically significant when *P*-values were less than 0.05.

## Results

### Patient characteristics

From July 2005 to December 2010, 94 white mCRC or patients with a locally advanced recurrence after surgery were included. Median age at the time of diagnosis was 63 years old. Their characteristics and UGT1A1 genotypes are shown in Table 1.

### Dose escalation, toxicities and DLT

The dose of irinotecan was escalated from 180 to 450 mg m^−2^ in ^*^1/^*^1 patients; from 110 to 390 mg m^−2^ in *1/^*^28 and from 90 to 150 mg m^−2^ in ^*^28/^*^28 (Table 2).

Table 3 summarises the total number of patients included at each dose level of irinotecan together with the number of cases with DLT.

In the group with the ^*^1/^*^1 genotype after two patients presented DLT (grade 3 diarrhoea at 220 mg m^−2^ and grade 3 asthenia at 260 mg m^−2^), six additional patients were treated with 260 mg m^−2^ and no DLT was observed. No patient presented a DLT in the 300 mg m^−2^ level. In the next level of doses (340 mg m^−2^), one out of six patients developed a DLT (grade 4 asthenia and grade 3 urinary tract infection plus fever causing hospitalisation). One out of six had a DLT (prolonged grade 4 asthenia and grade 3 neutropenia) at 390 mg m^−2^. One further patient at 390 mg m^−2^ level treated with only one dose of irinotecan was withdrawn because of a protocol violation. Finally, two out of five patients presented a DLT at 450 mg m^−2^. One patient presented grade 3 diarrhoea and vomiting plus a secondary grade 4 asthenia causing severe dehydration requiring intravenous fluids and hospitalisation. The other case of DLT at 450 mg m^−2^ consisted of grade 3 diarrhoea and asthenia. Therefore, the MTD in the group of patients with a ^*^1/^*^1 genotype was 390 mg m^−2^ (Table 3).

In the group with a ^*^1/^*^28 genotype no DLT was observed in the patients treated with less than 300 mg m^−2^. One patient presented a grade 4 neutropenia at 300 mg m^−2^ and one patient presented a grade 3 diarrhoea and asthenia at 340 mg m^−2^ level with catheter-related sepsis by *Staphylococcus aureus* requiring intravenous antibiotics and hospitalisation. This dose was the MTD in the ^*^1/^*^28 genotype patients because the two first patients treated with a dose of 390 mg m^−2^ experienced a DLT (grade 3 asthenia and persistent grade 3 neutropenia, respectively) (Table 3).

In the ^*^28/^*^28 genotype only one of six patients presented a DLT (grade 3 diarrhoea) at 90 mg m^−2^. No patient presented a DLT at the 130 mg m^−2^ level. As two of five patients at 150 mg m^−2^ developed a DLT, the MTD was established at 130 mg m^−2^. One patient presented grade 4 neutropenia and grade 3 asthenia and the other presented grade 3 asthenia and grade 3 constipation with intestinal subocclusion (Table 3).

Among all grade 3–4 toxicities, the most frequent severe haematological toxicity during cycle 1 was grade 3–4 neutropenia (25%). Grade 3–4 asthenia was the most common non-haematological toxicity (18%). Others severe toxicities were grade 3 diarrhoea (10%), infection without neutropenia (4%), nausea/vomiting (4%) and mucositis (1%). The different grade 3 to 4 toxicities according to the UGT1A1 genotype (including DLT and non-DLT) are summarised in Table 4.

### Effect of irinotecan dose on tumour response and survival

In all, 56 patients were assessable for tumour response. The overall response rate (ORR=complete plus partial response) was 46% (*n*=25). The ORR was 60% in patients with ^*^1/^*^1 genotype, 39% in those with ^*^1/^*^28 genotype and 13% in ^*^28/^*^28 (*P*=0.049).

To evaluate the relation between irinotecan dose and response rate, we grouped patients into two cohorts according to the median dose of irinotecan: 27 patients treated with ⩾260 mg m^−2^ and 29 patients treated with a <260 mg m^−2^. A statistically significant relationship between irinotecan dose and response rate was observed (*U*-test, Mann–Whitney; *P*=0.023). In all, 67% of patients treated with ⩾260 mg m^−2^ of irinotecan achieved a complete or partial response in comparison with only 24% of patients treated with <260 mg m^−2^ (*P*=0.001) (Table 5). In the logistic regression analysis only a ⩾260 mg m^−2^ irinotecan dose independently predicted the probability of response to FOLFIRI (odds ratio (OR)=5.71; CI 95%: 1.76–18.51, *P*=0.004). The dose level was also associated with a better response rate in the logistic regression analysis (*P*=0.02).

Median follow-up time was 13 months (range, 1–57 months). Median TTP was 10 months (range, 8–12 months). There were no differences in TTP according to the genotype (*P*=0.58). Median TTP was higher in patients treated with ⩾260 mg m^−2^ of irinotecan (16 months) than in patients treated with <260 mg m^−2^ of irinotecan (7 months) months (*P*=0.003).

In patients with assessable tumour response, a higher TTP was observed in those who achieved a complete or partial response (11 months) than in those with stable disease (10 months) or progressive disease (2.7 months; *P*<0.001) after FOLFIRI treatment. The Cox regression model included the UGT1A1 genotype, ECOG, sex, age, clinical-risk EORTC classification and primary tumour localisation. The risk-group classification (*P*=0.003; HR=1.95; CI 95%: 1.25–3.01) and a ⩾260 mg m^−2^ irinotecan dose (*P*=0.003; HR=2.9; CI 95%: 1.43–5.88) were independent predictors of TTP after adjustment for the other clinically relevant variables.

## Discussion

This study evaluated the DLT and MTD of irinotecan in the FOLFIRI regimen in first-line chemotherapy in mCRC according to UGT1A1 genotype. In this dose-escalating trial, we establish that the standard dose of 180 mg m^−2^ for irinotecan is significantly lower than the dose that can be tolerated by patients with a UGT1A1 ^*^1/^*^1 or ^*^1/^*^28 genotype. A dose escalation was evaluated for the first time in patients with a high-risk UGT1A1 ^*^28/^*^28 genotype, and we demonstrated that the MTD was 30% lower than the standard dose of 180 mg m^−2^. Although results of tumour response are exploratory, with this dose (130 mg m^−2^), irinotecan was poorly effective and a more active chemotherapeutic regimen should be considered for this group of patients.

This dose escalation trial of irinotecan in patients with UGT1A1 ^*^1/^*^1 and ^*^1/^*^28 genotypes led us to establish that 390 and 340 mg m^−2^, respectively, can be safely administered every 2 weeks (MTD) in mCRC patients undergoing first-line treatment with the FOLFIRI regimen. Though slightly higher, these doses, are very similar to those reported in a recent phase I study of irinotecan administered in the FOLFIRI regimen in Italian patients with mCRC: 370 mg m^−2^ for patients with UGT1A1 ^*^1/^*^1 genotype, and 310 mg m^−2^ for those with a ^*^1/^*^28 genotype. These authors identified these doses as safe to administer when mCRC patients were stratified according to the UGT1A1 genotype, and patients genetically at risk for toxicity (^*^28/^*^28) were excluded (Toffoli *et al*, 2010). One limitation of our study compared with this work is that a pharmacokinetics analysis was not carried out to describe the effect of different irinotecan doses on patient's drug exposure.

In Japan, the health authorities have approved a two-weekly dose of 150 mg m^−2^ for irinotecan monotherapy. A phase I/II study determined the recommended dose of FOLFIRI for patients with mCRC without the UGT1A1 ^*^28/^*^28 genotype. Although the MTD was not reached, the conclusions favoured an increase in the irinotecan dose for patients without the ^*^28 allele to 180 mg m^−2^ (Yamashita *et al*, 2011). In this same population, a phase I study of irinotecan and doxifluridine (5′-DFUR) for mCRC patients without UGT1A1 ^*^28/^*^28 genotype concluded that the recommended doses of biweekly irinotecan were 150 mg m^−2^ for patients with the UGT1A1 ^*^1/^*^1 genotype and 70 mg m^−2^ for those with the ^*^1/^*^28 genotype (Hazama *et al*, 2010). However, a recent Japanese study concluded that no significant differences in the efficacy or toxicity of FOLFIRI between patients with UGT1A1^*^1/^*^1 genotype and those with UGT1A1^*^1/^*^6 or ^*^1/^*^28 genotype, when irinotecan dose is 180 mg m^−2^ (Sunakawa *et al*, 2010). This suggest that when UGT1A1 ^*^28/^*^28 genotype is excluded, the dose of irinotecan is more relevant than the genotype.

In Caucasian populations, in Italy and Spain, the recommended doses in the phase I studies carried out to date are higher than those in the Japanese trials. These differences may perhaps be attributed to the ethnic differences in UGT1A1 genotypes and/or differences in the therapeutic regimens used. Other combinations with irinotecan that differ from the standard FOLFIRI regimen, include those using biological agents, should be explored before making any conclusions.

Our present results also show that a dose ⩾260 mg m^−2^ is an independent predictor of a better response and higher TTP in mCRC patients without the risk genotype (^*^28/^*^28). We therefore suggest that in future phase II and phase III studies, the initial dose of irinotecan in FOLFIRI regimen should be at least 260 mg m^−2^ in Caucasian patients who have either the ^*^1/^*^1 or ^*^1/^*^28 UGT1A1 genotype.

Finally, we suggest that as the MTD in patients with a high-risk UGT1A1 ^*^28/^*^28 genotype (130 mg m^−2^) is ineffective, other more active chemotherapeutic regimen should be considered for these patients.

## Figures and Tables

**Table 1:** Patients characteristics

	**No. of patients**		**Percentage**
*Sex*			
Male	57		61
Female	37		39
			
*Age median, years*		63	
Range		33–80	
			
*ECOG*			
0.1	87		93
2	7		7
			
*Risk group*			
Low	58		62
Intermediate	26		28
High	10		10
			
*Primary tumour*			
Colon	60		64
Rectal	34		36
			
*No of metastatic sites*			
1	63		67
⩾2	31		33
			
*Assessable*			
For safety		94	
For efficacy		56	
			
*UGT1A1 gene*			
^*^1/^*^1	42		45
^*^1/^*^28	38		40
^*^28/^*^28	14		15

Abbreviation: ECOG=Eastern cooperative oncology group.

**Table 2:** Irinotecan dose escalation according to the UGT1A1 genotype

	**Irinotecan dose (mg m** ^−**2**^ ) *1/*1	**Irinotecan dose (mg m** ^−**2**^ ) *1/*28	**Irinotecan dose (mg m** ^−**2**^ ) *28/*28
Level 1	180	110	90
Level 2	220	125	130
Level 3	260	150	150
Level 4	300	180	
Level 5	340	220	
Level 6	390	260	
Level 7	450	300	
Level 8		340	
Level 9		390	

**Table 3:** Dose escalation and DLT of increased Irinotecan doses in patients treated with FOLFIRI in ^*^1/^*^1, ^*^1/^*^28 and ^*^28/^*^28 patients

**Irinotecan Dose (mg m** ^−**2**^ **)**	No. of patients with *1/*1	**No. of patients with DLT**	**Type of DLT**	No. of patients with *1/*28	**No. of patients with DLT**	**Type of DLT**	No. of patients with *28/*28	**No. of patients with DLT**	**Type of DLT**
90	—	—	—	—	—	—	6	1	Grade 3 diarrhoea
110	—	—	—	3	0	—	—	—	—
125-130	—	—	—	3	0	—	3	0	Grade 3 asthenia
150	—	—	—	3	0	—	5	2	Grade 4 neutropenia. Grade 3 asthenia and constipation
180	3	0	—	9^a^	0	—	—	—	—
220	6	1	Grade 3 diarrhoea and nausea	3	0	—	—	—	—
260	12^a^	1	Grade 3 asthenia	3	0	—	—	—	—
300	3	0		6	1	Grade 4 neutropenia	—	—	—
340	6	1	Grade 4 asthenia. Urinary tract infection	6	1	Grade 3 diarrhoea. Sepsis by *Staph aureus*	—	—	—
390	6	1	Grade 4 asthenia	2	2	Grade 3 neutropenia Grade 3 asthenia	—	—	—
450	5	2	Grade 3 diarrhoea, vomiting and grade 4 asthenia. Grade 3 diarrhoea and asthenia	—	—	—	—	—	—

Abbreviation: DLT=dose-limiting toxicity.

a Six additional patients.

**Table 4:** First cycle grade 3/4 toxicities (including DLT and non-DLT)

	**Genotype**		
	*1/*1 *n*=41	*1/*28 *n*=38	*28/*28 *n*=14
**Toxicity**	***n* (%)**	***n* (%)**	***n* (%)**
Diarrhoea	4 (10)	2 (5)	3 (21)
Nausea/vomiting	2 (5)	2 (5)	0 (−)
Asthenia	10 (24)	4 (11)	3 (21)
Mucositis/stomatitis	1 (2)	0 (−)	0 (−)
Anorexia	0 (−)	0 (−)	0 (−)
Infection without concomitant grade III–IV neutropenia	2 (5)	0 (−)	1 (7)
Anaemia	0 (−)	0 (−)	0 (−)
Neutropenia	9 (22)	10 (26)	4 (29)
Thrombocytopenia	0 (−)	0 (−)	0 (−)
Fever with concomitant neutropenia	0 (−)	0 (−)	0 (−)

Abbreviation: DLT=dose-limiting toxicity.

**Table 5:** Response rate: effect of irinotecan dose and UGTA1 genotype

		**CR+PR**				**SD+PD**				
**Response**	***n***	***n***	**Percentage**	**CR**	**PR**	***n***	**%**	**SD**	**PD**	***P***
Overall	56	25	46	4	21	31	54	22	9	
^*^1/^*^1	25	15	60	1	14	10	40	7	3	0.049
^*^1/^*^28	23	9	39	3	6	14	61	8	4	
^*^28/^*^28	8	1	13	–	1	7	87	5	2	
⩾260 mg m^−2^	27	18	67	2	16	9	33	8	1	0.001
<260 mg m^−2^	29	7	24	2	5	22	76	14	8	

Abbreviations: CR=complete response; PD=progressive disease; PR=partial response; SD=stable disease.
